# Strain-Affected Hydrogen Diffusion Under Biaxial Stress in α Iron

**DOI:** 10.3390/ma19030486

**Published:** 2026-01-26

**Authors:** Zhiqin Du, Zhonghao Heng, Jian Li, Chen Jin, Jianghua Shen

**Affiliations:** 1 School of Aeronautics, Northwestern Polytechnical University, Xi’an 710072, China; doogon@mail.nwpu.edu.cn (Z.D.); j.shen@nwpu.edu.cn (J.S.); 2 School of Mechanical Engineering, Qinghai University, Xining 810016, China; lijian1979@qhu.edu.cn; 3 Qinghai Salt Lake Trimagnesium Co., Ltd., Xining 811600, China; jinchen@yhtrimag.cn; 4 Shaanxi Key Laboratory of Impact Dynamic and Its Engineering Application, Northwestern Polytechnical University, Xi’an 710072, China

**Keywords:** α iron, hydrogen diffusion, biaxial stress, molecular dynamics

## Abstract

A deep understanding of hydrogen diffusion in metals under stress is crucial for revealing the mechanism of hydrogen embrittlement. While the effects of isotropic and uniaxial stress have been studied, the atomic-scale mechanism under a pure biaxial stress state remains unclear. This work employs molecular dynamics simulations to investigate hydrogen diffusion in α-iron under controlled biaxial stress. The results show that biaxial stress influences diffusion indirectly by altering the lattice geometry and thus the migration energy barrier. It is found that the diffusion path is governed by the direction of the minimum principal strain, while the diffusion rate is controlled by the maximum tensile principal strain, with which it exhibits an approximately exponential relationship. These insights clarify the distinct roles of different strain components, providing a refined framework for understanding hydrogen behavior under complex stress states and guiding the design of hydrogen-resistant materials.

## 1. Introduction

Hydrogen energy is widely used in industries such as transportation, power generation, and construction, and is recognized as a crucial pathway for deep decarbonization [1,2,3]. However, a key challenge to its safe application is hydrogen embrittlement (HE) in metallic storage and structural components. This phenomenon is fundamentally linked to the diffusion of hydrogen atoms within the metal lattice [4,5,6]. Although stress is a well-established driver for hydrogen diffusion and accumulation, a critical gap remains in understanding its underlying atomic-scale mechanisms. Existing research has firmly established the influence of isotropic stress on hydrogen diffusion [7,8,9,10], and constitutive laws based on hydrostatic stress gradients have been developed [11]. However, these models show significant limitations in explaining the anisotropy of stress-induced hydrogen diffusion that has been observed [12,13]. Subsequent studies have thus explored various anisotropic stress states, seeking to relate stress to changes in diffusion activation enthalpy. Nonetheless, these investigations have largely been confined to simple uniaxial or shear stress states, often lacking direct connection to specific, service-relevant conditions. A prominent example is the pure biaxial stress state, which is a common loading condition in thin-walled pressure vessels and membranes [14]. Its specific role in governing atomic-scale hydrogen diffusion mechanisms remains almost entirely unexplored.

To bridge this knowledge gap, molecular dynamics simulations were employed to directly investigate hydrogen diffusion in α-iron under controlled biaxial stress. This study aims to clarify the underlying mechanism of how biaxial stress influences hydrogen behavior by examining its effects on atomic diffusion trajectories, diffusion rates, and key controlling factors. The findings offer fundamental insights for the design of more reliable hydrogen-resistant materials.

## 2. Methodology

**Figure 1:**
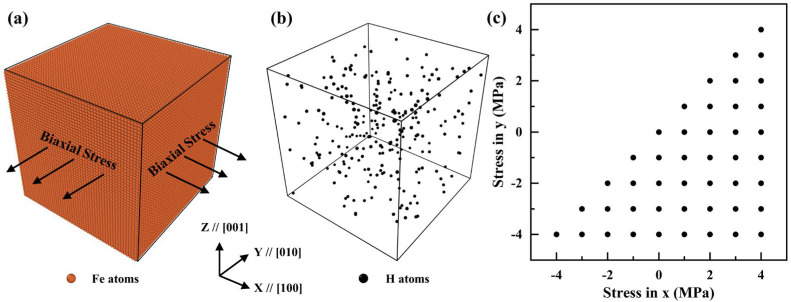
Schematics of diffusion simulation. (**a**) Biaxial stress is applied in the x–y plane. (**b**) Hydrogen atoms distribute randomly in a α iron lattice. (**c**) According to the symmetry, biaxial stress is applied with 45 states (represented as black dots).

Molecular dynamics (MD) simulations are conducted in a Fe-H system using LAMMPS (version 27 Jun 2024) [15] and the results are visualized using OVITO (version 2.9.0) [16]. The atomic interaction is described using the potential developed by Wen [17]. The simulation box is established in a size of $50\;a_{0}\times 50\;a_{0}\;\times 50\;a_{0}$, where $a_{0}$ is the lattice constant of α iron. The x, y, and z directions are all set in periodic boundary condition and corresponding to the [100], [010], and [001] directions, respectively. Hydrogen atoms are randomly added to the Fe lattice with concentration of 0.1 at. %. The timestep of MD simulation is set as 1 fs. To reach a lower energy state, the models performed energy minimization through the conjugate gradient method and were relaxed at 300 K for 10 ps with an isothermal-isobaric ensemble. To investigate the effect of biaxial stresses on hydrogen diffusion, pressures ranging from −5 to 5 GPa are applied on the x–y plane through Nosé-Hoover thermostat with 45 stress states as shown in Figure 1. The z direction, normal to the loading plane, is an unstressed direction. The diffusion simulation covers a total of 100 ps under a temperature of 300 K. The diffusion coefficient, namely diffusivity, is calculated by Einstein’s equation using Equation (1):

(1) $$D=\frac{<R^{2}>}{2nt}$$

where $<R^{2}>$ is the mean square displacement of the hydrogen atoms, n is the dimension of the system, and t is the simulation time. The effectiveness has been demonstrated in many studies [18,19,20,21,22].

## 3. Results and Discussion

**Figure 2:**
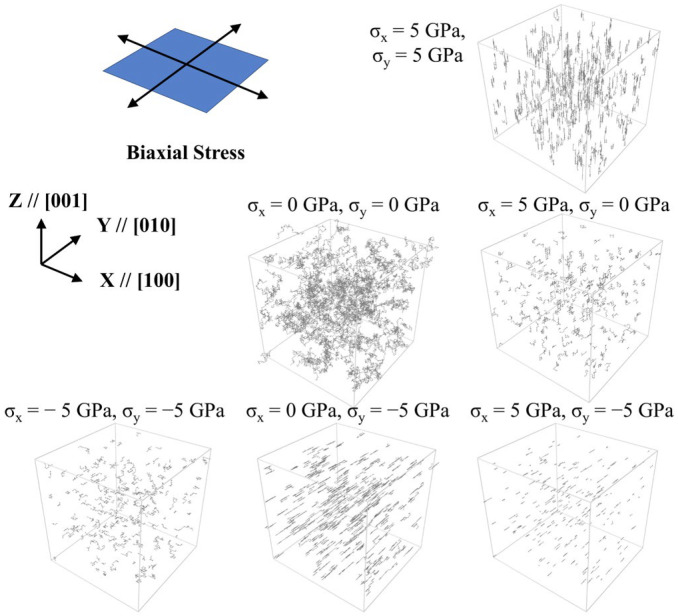
Trajectories of hydrogen atoms under different stress states.

The trajectory lines of hydrogen atoms are shown in Figure 2. Under uniaxial compression stress, the diffusion in the loading direction is significantly faster than that in the unstressed direction, and hydrogen atoms almost only diffuse in a single direction. Under uniaxial tensile stress, the diffusion in all directions is relatively uniform, but slower than that without stress. The result in uniaxial state is consistent with our previous work [23] that applied a different potential, which emphasizes the credibility. Under biaxial tensile stress, hydrogen atoms diffuse along a single direction again, but the direction is normal of the loading plane with no stress loading. For the biaxial state with one tensile and the other compressive, hydrogen atoms diffuse along the compression direction, but significantly slower than the cases of uniaxial compression and biaxial tension. Unidirectional diffusion occurs in both uniaxial compression and biaxial tension states, and limited diffusion occurs in uniaxial tension and biaxial compression states, implying the existence of some commonality that affects the direction and rate of diffusion in these situations.

**Figure 3:**
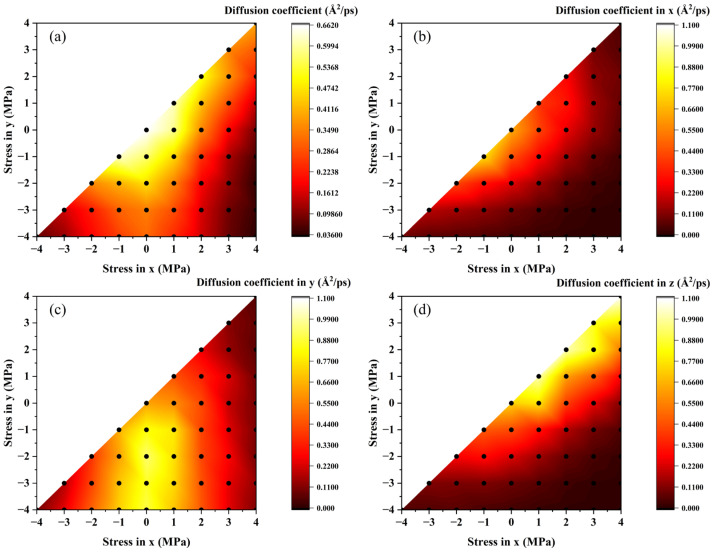
The relationship between diffusion coefficient and biaxial stress: (**a**) three-dimensional diffusion coefficient, and (**b**–**d**) one-dimensional diffusion coefficient in the x, y, and z directions. Black dots represent original data points. (For interpretation of the references to color in this figure legend, the reader is referred to the web version of this article).

The relationship between biaxial stress and diffusion coefficient is shown in Figure 3a. In the unstressed sample, the diffusion coefficient is the highest, and any increase in tensile or compressive stress will lead to a decrease in the diffusion coefficient. It can be found that the diffusivity of uniaxial tension is lower than that of uniaxial compression, that is, the effect of a tensile state is more significant than a compressive state. This is consistent with the findings of Nagase et al. [13] and us [23], which confirms the credibility of this work. The diffusivity may be similar under different stress states, but combining with the trajectory lines in Figure 2, the preferred direction of diffusion might be inconsistent. One-dimensional diffusivity is calculated as shown in Figure 3b–d. It can be found that under biaxial stress conditions, an increase in compressive stress in one direction is beneficial for the diffusion of hydrogen atoms in that direction. In addition, diffusivity in the unpressed direction is also affected by biaxial stress. If there is compressive stress in any direction in the loading plane, the diffusion in the normal direction will be suppressed. Notably, some models show quite different diffusion characters though their hydrostatic stress is the same, indicating the limitation of evaluating diffusion based on isotropy.

**Figure 4:**
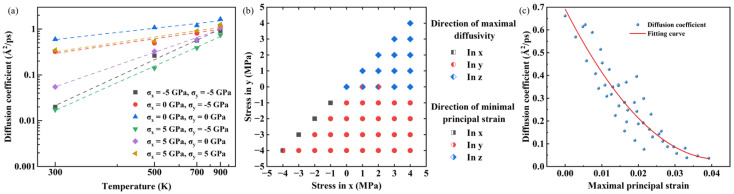
(**a**) The relationship between temperature and diffusion coefficient; (**b**) the direction of the minimum principal strain and the direction of the maximum one-dimensional diffusion coefficient; (**c**) the relationship between maximum principal strain and diffusion coefficient. (For interpretation of the references to color in this figure legend, the reader is referred to the web version of this article).

Because of the complexity of the effect of biaxial stress on hydrogen diffusion, a more concise theory is required. Additional simulations were performed calculating the diffusion coefficients under different biaxial stress states at temperatures ranging from 300 to 900 K, as shown in Figure 4a. It can be found that hydrogen diffusion still conforms to the Arrhenius relationship, which means that it remains a thermal process under biaxial stress. Meanwhile, significant differences exist in the slopes of these curves, indicating that the effect of stress on hydrogen diffusion is due to its change in activation energy. Classical continuum models describe hydrogen diffusion by linking the hydrogen flux to gradients in hydrostatic stress [11]. However, since the resulting hydrostatic strain is isotropic, it cannot account for directional effects, highlighting the need for alternative metrics to quantify the strain-diffusion relationship. Here, the principal strain is considered. That is to say, the change in diffusion coefficient is due to the alteration in migration potential barrier influenced by the lattice geometry. In other words, the diffusion of hydrogen atoms is directly related to the strain instead of the biaxial stress. Owing to the Poisson effect, the strain state of the model is more complex than the stress state. The applied stress is on a plane, but the strain state is three-dimensional. The strain in all three directions is principal strain, which is because of the absence of applied shear stress. Therefore, the principal strain is calculated according to Equation (2):

(2) $$\varepsilon_{i}=\frac{∆l_{i}}{l_{i}}$$

where i is a direction (x, y or z), $l_{i}$ is the length of the model in this direction, and $∆l_{i}$ is the increment of it. According to the Poisson effect, biaxial tensile strain can cause compressive strain in the normal direction, and biaxial compressive strain cause tensile strain, conversely. As mentioned earlier, unidirectional diffusion in the normal direction occurs under biaxial tension. Combining with other cases of unidirectional diffusion, it can be noted that unidirectional diffusion occurs in the direction of strain compression. Therefore, it is inferred that the preference of diffusion direction is related to compressive strain. Due to the biaxial stress state, the principal strain is compressed in at least one direction, meaning that the minimum principal strain can represent the compressed portion of the corresponding stress state. Figure 4b shows the correspondence between the direction of the minimum principal strain and the direction of the maximum diffusivity in all data points. It can be observed that these two directions have a highly consistent correspondence. Therefore, the inference that compressive strain determines diffusion tendency has been confirmed. Furthermore, as mentioned earlier, it is believed that tensile strain is more related to diffusivity than compressive strain. Figure 4c shows the relationship between maximum principal strain and diffusion coefficient. It can be found that the larger the principal strain, the smaller the diffusion coefficient, and there is an approximate exponential relationship between the two parameters. The larger the principal strain, the smaller the diffusion coefficient, and it gradually approaches 0. This indicates that a tensile state has a greater impact on the potential barrier than a compressive state. Differing from the consideration of activation enthalpy [13], this work directly establishes the relationship between strain and diffusion, facilitating a more direct discussion of the effects of biaxial stress. Furthermore, these findings provide an enlightening significance in engineering that for pressure vessel wall materials, which works in the biaxial stress state, the hydrogen diffusion inside can be suppressed through pre-loading stress.

## 4. Conclusions

In summary, this work advances the understanding of hydrogen diffusion in two key aspects. First, it confirms that under biaxial stress, hydrogen diffusion remains a thermally activated process. The observed changes in the diffusion coefficient are attributed to the influence of biaxial stress on the lattice geometry, which subsequently alters the migration energy barrier. More importantly, it is discovered that the diffusion path and the diffusion rate are governed by different components of the strain tensor: the direction of minimum principal strain selects the preferred diffusion path, while the magnitude of the maximum tensile strain controls the rate. Specifically, the diffusion coefficient exhibits an approximately exponential relationship with the maximum tensile principal strain. These mechanistic insights provide a more refined framework for understanding and predicting hydrogen behavior under complex stress states.

Collectively, these insights establish a fundamental framework for understanding hydrogen diffusion at the atomic scale under the previously underexplored biaxial stress condition. Looking forward, incorporating the influence of microstructural defects (e.g., vacancies, grain boundaries) under stress will be essential to predict hydrogen behavior in real engineering materials and to guide the design of advanced hydrogen-resistant alloys.

## Data Availability

The original contributions presented in this study are included in the article. Further inquiries can be directed to the corresponding author.
